# Pediatric Pleural Effusion and Pneumococcal Vaccination Trends in the Pre- and Post-COVID Era: A Single-Centre Retrospective Study

**DOI:** 10.3390/children12020242

**Published:** 2025-02-18

**Authors:** Denisa Lavinia Atanasiu, Maria Mitrica, Luciana Petrescu, Oana Falup-Pecurariu, Laura Bleotu, Raluca Ileana Lixandru, David Greenberg, Alexandra Grecu

**Affiliations:** 1 Emergency Clinical Hospital for Children Brasov, Brasov 500063, Romania; denisalavinia@gmail.com (D.L.A.); f-med@unitbv.ro (M.M.); secretariat.scbv@gmail.com (L.P.); oana.falup_pecurariu@unitbv.ro (O.F.-P.); laurableotu@gmail.com (L.B.); mariana.lazar@unitbv.ro (A.G.); 2 Faculty of Medicine, Transylvania University of Brasov, Brasov 500036, Romania; 3 Soroka Medical Centre, Ben Gurion University, Beer-Sheva 8410501, Israel; dudi@bgu.ac.il

**Keywords:** pleural effusion, pediatric empyema, post-COVID-19 immunity, pneumococcal vaccination

## Abstract

**Background/Objectives**: Pleural effusion represents an accumulation of fluid in the pleural cavity, frequently associated with pneumonia. There has been a gradual increase in cases among children in recent years, with a notable rise during the post-pandemic period, potentially due to immune debt, decreased vaccination coverage, and changes in pathogen dynamics. **Methods**: We enrolled 66 children with pleural effusion treated at the Children’s Emergency Clinical Hospital, Brasov, between January 2019 and September 2024. We analyzed the data on demographics, symptoms, vaccination status, hospitalization, and treatments to assess the trends in the incidence and clinical features. **Results**: The median age was 5 years (ranging from 3 months to 17 years). Most patients were male (57.5%) from rural areas (34.8%). Only 40.9% fulfilled the vaccination schedule of Romania. We observed a rise in hospitalizations in the last two years, with 16 cases in 2023 and 15 in 2024, and most were being admitted in April (15.5%). Patients mainly had severe (36%) and medium (26%) acute respiratory failure. *S. pneumoniae* was the most common isolate with two cases each of serotype 1, 14, and 23A, and one case each of serotype 3, 31, and 34, followed by *H. influenzae* and *P. aeruginosa*. Treatment was mostly with ceftriaxone (69.6%), Vancomycin (63.6%), Meropenem (53.0%), and Teicoplanin (25.7%). Some children required thoracic drainage (34.8%). Complications like pneumothorax (16.6%), polyserositis (4.5%), and pneumomediastinum (3.0%) were found. **Conclusions**: The rise in pleural effusion cases may be influenced by various factors, such as changes in pathogen behavior or host immune responses following the pandemic. Further research is needed to understand these potential mechanisms. The emergence of non-PCV20 strains and the common occurrence of serotype 3 infections point out the need to study serotype trends and evaluate whether expanding vaccine programs could be beneficial.

## 1. Introduction

Pleural effusion is an accumulation of excess fluid in the pleural cavity [1]. It is frequently associated with pneumonia in the pediatric population and is more common in younger male children [2].

The incidence of empyema and pleural effusion in children has been rising in industrialized countries, although the rates vary considerably across regions. In the United States, cases grew from 2.2 per 100,000 in 1997 to 3.7 per 100,000 in 2006 [3]. Other studies have reported dramatic surges: an eight-fold increase in Spain between 1999 and 2004 and an almost twenty-six-fold spike in France between 1995 and 2003 [2]. We did not have enough data to determine the incidence of pleural effusion among children in Romania. Moreover, there are scarce data regarding pleural effusion pre- or post-COVID-19 in Romanian children.

The PCV has been shown to help reduce IPD and the associated complications: a 12-year cohort study in Italy showed significant reductions in IPD in those vaccinated with PCV compared with those not vaccinated. For children born before 2011, the crude incidence rate was 2.82 per 1,000,000 person-years, and the age-standardized rate was 0.66 per 1,000,000 person-years for children born after 2011 and vaccinated with PCV13. This research also showed that the real-time PCR testing of pleural fluid is an essential tool for the better identification of the causes of PPE and monitoring vaccination programs [3].

A study from the Netherlands reported approximately 1,016,685 respiratory disease hospitalizations among children under 5 years old in the EU-28 and Norway. Romania had one of the highest hospitalization rates, with 123,377 cases [4].

The COVID-19 pandemic has greatly disrupted the access to health services, vaccination programs, and infection prevention protocols, which may have affected not only the incidence but also the temporal patterns of pleural effusion among children. The implementation of social distancing and mask-wearing practices may alter the exposure to common pathogens, potentially affecting the epidemiology of respiratory diseases and related complications, such as pleural effusion.

## 2. Materials and Methods

We conducted an observational retrospective study in which we enrolled 66 patients hospitalized for pleural effusion at the Brasov Children’s Emergency Hospital, in the PICU from January 2019 to September 2024. We considered pleural effusion in patients with chest X-rays that presented with pleural fluid, according to the WHO case definition. The study included patients with both complicated pleural effusion (empyema), as well as those with uncomplicated pleural effusion. The vaccination status of the patients was verified through the RENV platform (National Electronic Vaccination Registry).

We evaluated demographics, vaccination status, season and year of hospitalization, length of hospitalization, clinical presentation, laboratory findings, sites of infection, and antibiotic treatment.

At the last census in 2021, Brasov County recorded a total population of 546,600 people, with approximately 263,800 people living in the city of Brasov. The pediatric population was approximately 116,000 people [5]. In 2022, the county had a birth rate of 5421, while the city of Brasov had 2494 of these births [6].

We present below the total number of ED presentation and the total number of admissions in the Acute Therapy Department by year (Table 1).

Nasal and oropharyngeal swabs and laryngotracheal secretion were inoculated within 2 h of collection. Plating was performed on Columbia agar medium with 5% defibrinated ram blood, with dispersion in 4 quadrants. Incubation of the plates was carried out for 48 h at 37 °C in a 5% CO_2_ environment.

Sediments with no purulent fragments were centrifuged and cultured. After plating, the samples were placed on various media, such as Columbia Agar 5% defibrinated ram blood, chocolate agar with Vitox, lactose medium, and Sabouraud agar with Chloramphenicol, with dispersion in 4 quadrants. Two smears were prepared and stained using Gram and/or Giemsa stains.

Pleural fluid was processed as soon as possible after arrival at the laboratory. It was homogenized and 1–2 mL was inoculated into both aerobic and anaerobic blood culture bottles. Elemental counts were performed on the hematology analyser, set for biological fluids. Samples were centrifuged at 3500 RPM for 10–15 min. Biochemical tests were performed on the supernatant. From the sediment, samples were inoculated on the same medium and dispersed in 4 quadrants, similar with laryngo-tracheal secretions, but 4 smears were prepared instead of 2. As with the previous ones, samples were identified on MALDI-TOF or Vitek and antibiograms were performed by the diffusimetric method or the breakpoint method on Vitek. All these samples were incubated for 48 h at 37 °C and evaluated at 24 h and 48 h. Pneumococcal serotyping was performed at Ben Gurion University, Soroka Medical Centre, Israel.

Multiplex quantitative real-time PCR or Biofire Filmarray rapid PCR assays were performed on lower respiratory tract secretions and pleural fluid samples. The Biofire Pneumo Plus panel identifies bacterial pathogens reported at 10^4^, 10^5^, 10^6^, or ≥10^7^ copies/m, and performs qualitative detection of atypical bacteria, viruses, and antimicrobial resistance genes.

At the Children’s Clinical Hospital, presumptive identification of *S. pneumoniae* was based on α-hemolysis and optochin inhibition, confirmed by positive slide agglutination. Each *S. pneumoniae* colony was subcultured, harvested, and frozen at −70 °C for further analysis.

Pneumococcal serotyping was conducted on each isolate at the Pediatric Infectious Disease Unit, Soroka University Medical Center, located in Beer-Sheva, Israel, using antisera produced by the Statens Serum Institut.

## 3. Results

Thirty-eight (57.5%) were male and twenty-eight (42.5%) were female. Their ages ranged from 3 months old to 17 years and 8 months old (mean age: 5 years old). Half of them (50%) were between 0–2 years old. Regarding the ethnicity of the population, twenty-seven (41.0%) were Romanian children, twenty-three (34.8%) Roma, and sixteen (24.2%) Hungarian. Thirty-eight of them (57.5%) came from rural areas. Twenty-seven children (40.9%) were fully vaccinated, while twenty (30.3%) received partial vaccination and nineteen (28.8%) had no vaccine.

**Figure 1:**
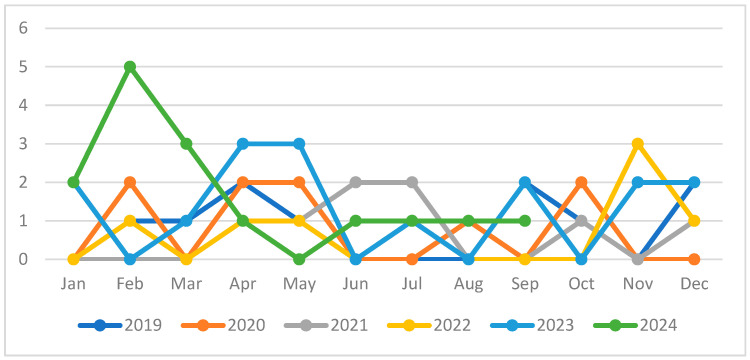
Distribution of pleural effusion cases by month of the year.

In 2019, ten children with pleural effusion were admitted to the ward. The number dropped to nine in 2020, then dropped to eight in 2021, and, in 2022, there were still eight. These subsequently increased significantly to sixteen hospitalizations in 2023 and fifteen in 2024. In total, nineteen children were hospitalized in winter (28.7%), twenty-three in the spring (34.8%), ten in the summer (15.1%), and fourteen in the autumn (21.2%). The graph below represents the distribution of cases by month of the year at the time of admission (Figure 1).

The data indicate that pediatric hospitalizations revealed a seasonal pattern, with the highest frequency occurring in April (15.5%), followed by February (13.6%) and May (12.1%). On the other side, the lowest incidence was observed during the months of June (4.5%) and August (3.0%).

Based on the child population of Brasov and the number of pleural effusion cases in our hospital, the annual incidence per 100,000 individuals was observed to be 8.62 in 2019, 7.76 in 2020, 6.90 in both 2021 and 2022, 13.79 in 2023, and 12.93 up to the present in 2024.

All cases had community-acquired pneumonia which led to pleural effusion. The duration of hospitalization ranged from 1 to 52 days (mean of 16.7 days) and the most of patients were hospitalized between 7 to 14 days. The study found that the length of hospital stay was closely associated with the children’s vaccination status: fully vaccinated individuals had an average hospital stay of 14.3 days, while those who were incompletely vaccinated or unvaccinated had an average stay of 18.3 days.

Most of the children were previously healthy. Three children had a SARS-CoV2 co-infection in the moment of admission, one had type A flu, and another one was convalescent after the same disease. One of the patients was diagnosed with measles.

Seventeen (25.7%) had chronic underlying illnesses. Eight patients (12.1%) were known to have cardiac disease, five (7.5%) presented intellectual disabilities, two (3%) had a former history of meningitis, one patient (1.5%) has been diagnosed with bronchopulmonary dysplasia at birth, and another patient (1.5%) has been known to have Prader–Willi syndrome.

**Figure 2:**
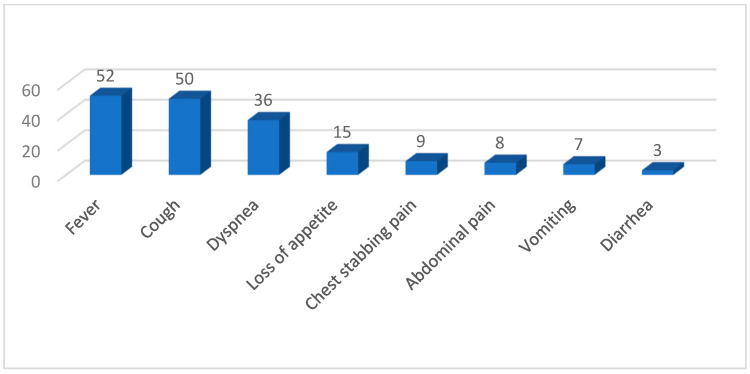
Clinical features observed in our study population.

The clinical manifestations identified in our study cohort are presented in Figure 2.

Patients suffered mainly from severe and medium acute respiratory failure—respectively, twenty-four (36%) and seventeen (26%) cases. Only in the last two years did we record seven cases each. A higher number of children who received the full vaccination schedule had no respiratory failure, while the incidence of moderate or severe respiratory failure was not significantly affected by vaccination status.

Thirty-two patients (48.4%) had significantly elevated WBC counts (ranging from 760 to 67,000/dL; mean of 18,739/dL). The majority had neutrophilia.

The patient’s inflammatory markers were measured, showing the highest average values in 2020: ESR 78.62 mm/h, CRP 26.51 mg/dL, and Fibrinogen 719.83 mg/dL, followed by 2022: ESR 76.71 mm/h, CRP 19.26 mg/dL, and Fibrinogen 677 mg/dL.

The initial chest X-rays for some of our patients did not suggest pleural effusions, but later radiographic studies revealed them. Twenty-five cases (37.9%) involved the right lung, twenty-nine (44%) involved the left lung, while twelve (18.1%) had both lungs affected. A confirmatory CT of the chest was performed in twenty-one (31.8%) cases.

In thirty children, at least one pathogen was identified. Seven patients (10.6%) had a positive pleural fluid culture, and one (1.5%) had positive pleural fluid PCR, while thirteen children (19.7%) had a positive blood culture, six (9.0%) had positive blood PCR, seven (10.6%) had positive laryngotracheal secretion, and eight (12.1%) were positive in respiratory PCR. We also found eleven (16.6%) positive nasal swabs and two (3%) positive oropharyngeal swabs. *S. pneumoniae* was the most common isolate, identified in nineteen patients, followed by *H. influenzae* in six individuals and *P. aeruginosa* in three individuals. Other bacteria included Coagulase negative *Staphylococci*, *S. pyogenes*, *M. catarrhalis*, *K. pneumoniae*, *K. oxytoca*, *S. viridans,* and MRSA. Twelve polymicrobial cases were identified. Two patients with positive QuantiFERON-TB Gold tests were diagnosed with tuberculous pleural effusion. No anaerobic bacteria were isolated. Regarding other types of pathogens, we identified four cases of Rhinovirus, one case of Parvovirus B19, and one case of *Candida albicans*.

Table 2 provides the demographic data and results of all cultures and PCRs.

**Figure 3:**
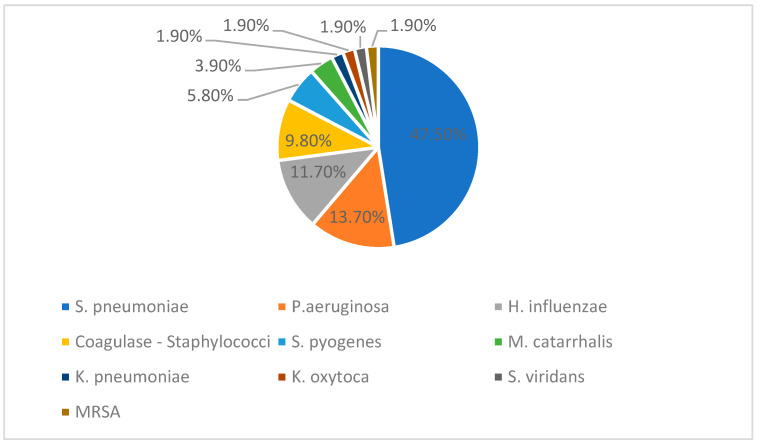
Bacteria results from cultures and PCR.

The figure below (Figure 3) represents the percentage distribution of bacterial isolates reported among the total of fifty-one positive cultures. Twenty-four positive cultures included *S. pneumoniae*, six were *H. influenzae*, seven were *P. aeruginosa*, and then five Coagulase negative *Staphylococci*, three *S. pyogenes*, two *M. catarrhalis*, and one each *K. pneumoniae*, *K. oxytoca*, *S. viridans,* and MRSA.

We identified only nine serotypes of pneumococcus, as only samples from the last two years were sent abroad for serotyping. Out of the 114 pneumococcal total samples collected, all of them survived transport and were serotyped according to the methods described previously. A significant part of them originated from patients hospitalized in other departments. The serotyping of pneumococci revealed two cases each of serotype 1, 14, and 23A, and one case each of serotype 3, 31, and 34.

Treatment with empirical broad-spectrum parenteral antibiotics was initiated after the appropriate cultures were collected. Antibiotic regimens were later modified based on clinical evolution, inflammatory markers, and culture results. Our patients went under treatment with ceftriaxone (69.6%), Vancomycin (63.6%), Meropenem (53.0%), and Teicoplanin (25.7%). Macrolides, aminoglycosides, quinolones, penicillins, and other antibiotic classes were rarely used.

Twenty-three children (34.8%) required thoracic drainage. Chest tubes were placed on admission (before antibiotic therapy commenced) or during the hospitalization. Six patients each from 2020 and the last two years, three patients in 2019, and two patients in 2021 had chest tubes inserted. Regarding the macroscopic appearance, ten children (47.6%) had yellow-citrine pleural fluid; in six children (28.6%), it was purulent; and, in five (23.8%), it was hemorrhagic.

Imaging studies of one child revealed pulmonary abscesses and a right chest wall abscess. A right thoracotomy was performed, which involved the evacuation of the chest wall abscess, pleuropulmonary decortication, and passive pleural drainage. Three patients were intubated and cared for in the pediatric intensive care unit. One of these patients undergoing pleural drainage developed severe bronchospasm with desaturation to 40% and sinus bradycardia progressing to asystole. Cardiopulmonary resuscitation maneuvers were effective in restoring central and peripheral pulses, and orotracheal intubation was then performed. Three others died from cardiorespiratory arrest.

**Figure 4:**
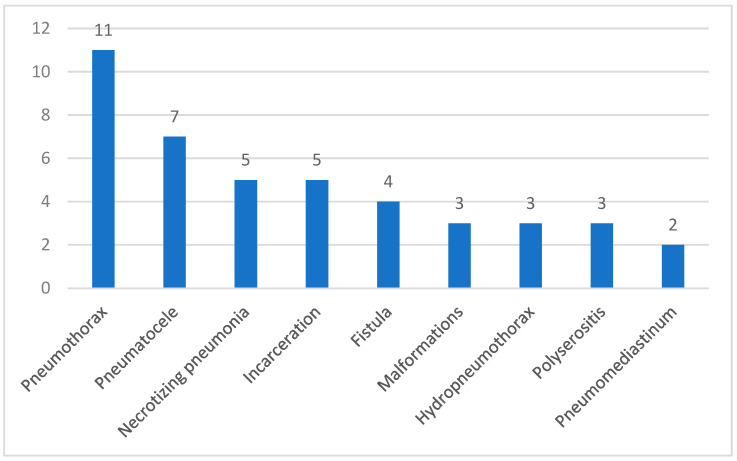
Complications following pleural effusion.

A total of 23 patients (34.8%) developed one or more of the complications presented in Figure 4. Six patients were fully immunized, while seventeen patients were either incompletely vaccinated or lacked vaccination.

## 4. Discussion

In our study, we found that around half of the cases are in male children, coming from rural areas, aged between 0–2 years, findings that are consistent with other published data [2].

Seasonal respiratory viruses such as influenza and respiratory syncytial virus can weaken the immune system, creating conditions for bacteria to thrive. Restrictive measures implemented during the COVID-19 pandemic, such as lockdowns, masking, and social distancing, may have diminished children’s exposure to infectious agents, potentially reducing their adaptive immunity, which could have made them more vulnerable to severe bacterial infections [7,8]. The pandemic also reduced the adherence to IPV, affecting children’s natural development of immune defences and their ability to fight infections [9].

Over the past two years, there has been a notable increase in the incidence of severe and complicated pleural effusions. This trend could be associated with the rise in viral infections following the pandemic. However, the specific relationship between these factors needs further investigation.

In 2022, North America and Europe experienced a significant rise in pneumonia cases among the pediatric population, followed by China with a similar pattern a year later. Researchers believe that the reappearance of influenza virus and RSV epidemics may have contributed to these substantial increases in cases [10].

Less than a half of the patients were fully vaccinated with PCV13 (1, 3, 4, 5, 6A, 6B, 7F, 9V, 14, 18C, 19A, 19F, and 23F), although it is on the Romanian NIP. PCV20 (1, 3, 4, 5, 6A, 6B, 7F, 8, 9V, 10A, 11A, 12F, 14, 15B, 18C, 19A, 19F, 22F, 23F, and 33F) is not in the NIP and has been approved for pediatric use in the EU since March 2024. Considering that proper vaccination can prevent serious complications like empyema, necrosis, fistula, and lung abscesses [11], it is easy to understand why so many children had multiple complications during their hospital stay.

The most common detected pathogens were *S. pneumoniae*, followed by *H. influenzae* and *P. aeruginosa*. These findings match previous studies, which show that *S. pneumoniae* remains the primary cause of PPE and empyema, despite pneumococcal vaccination efforts. The continued presence of *H. influenzae* and *P. aeruginosa* emphasizes the need for close microbiological monitoring and targeted antibiotic treatment.

Pneumococcal vaccine coverage has an alarming decline in our country, Romania. The vaccination rate at 12 months old decreased from 75% in 2018 to 59% in 2022. At 18 months old, it decreased from 85% in 2018 to 78% in 2021. At 24 months old, it decreased from 88% in 2018 to 82% in 2021 [12]. Of the nineteen patients with a positive culture of pneumococcus, only five were fully vaccinated, seven had received only one dose of PCV13, and another seven had none.

This study identified *S. pneumoniae* serotypes 1, 3, 14, 23A, 31, and 34 in patients, showing us a problem of invasive pneumococcal disease despite vaccination efforts. While PCV13 has significantly reduced infection rates, certain serotypes (especially serotype 3) continue to cause breakthrough infections, such as pleural effusion and empyema, especially in young individuals [13]. We did not see an important serotype replacement in our patients presumably due to a low coverage of the vaccine.

The use of pneumococcal vaccines has reduced the severity and deaths caused by IPD [14]. However, even with the broad coverage of PCV13, some of our identified strains were not targeted by the vaccine. This suggests that, while the vaccine serotypes have decreased, other non-vaccine strains have become more common, likely due to serotype replacement [15].

Our study did not find any PCV20-PCV13 *S. pneumoniae* serotypes for empyema patients, but research indicates that serotypes 11A, 15B, 22F, and 33F are commonly encountered and associated with antibiotic resistance to beta-lactams, macrolides, cephalosporins, and TMP-SMX [16].

Following a meta-analysis, it was discovered that PCV13 has been shown to be effective against IPD with serotype 3 in children, but it is unclear whether a booster dose confers greater protection. Some studies have shown that the immune response to serotype 3 after the booster dose was not modified. Pneumococcal vaccination also reduces the number of carriers and, thus, provides indirect protection against the disease [17].

It is important to reduce the risk in both healthy children and those with underlying conditions, so future pneumococcal vaccines must target these emerging serotypes and improve the protection against resilient ones like serotype 3.

A German study highlighted that aminopenicillins, beta-lactamase inhibitors, are the first-line treatment for pleural empyema in children [18]. In France, intravenous amoxicillin/clavulanic acid monotherapy was predominantly used, unless signs of severity were present [19].

In contrast, in our study, a greater tendency was observed in using third-generation cephalosporins, which are recommended in areas with a high incidence of penicillin-resistant *S. pneumoniae* [20].

It has been reported that vancomycin is recommended in areas with many MRSA carriers. Although guidelines recommend piperacillin–tazobactam and clindamycin for children with underlying conditions [20], these antibiotics were seldom used in our cohort.

No significant difference was found in the literature between monotherapy and combination therapy, suggesting that aggressive treatment with broad-spectrum antibiotics may not improve outcomes [18].

Aminoglycosides have low efficacy due to their inactivation in pleural fluid [20].

This may reflect the differences in local antibiotic resistance patterns, clinical practice, or the specific pathogen profile of our patient population.

## 5. Conclusions

This study highlighted a worrying increase in cases of pleural effusion among the pediatric population in our hospital, especially in vulnerable categories (underprivileged young children). This result might be associated with changes in the immune system, potentially influenced by pandemic-related restrictions, the decreased exposure to infectious pathogens, and the lower vaccine coverage. Further research is needed to better understand these relationships.

*Streptococcus pneumoniae* remains the predominant pathogen in our cohort, with serotype 3 being frequently identified, even among vaccinated individuals. In addition to the low vaccination rate, the emergence of non-vaccine serotypes further complicates the management of the disease. It could be beneficial to raise the vaccination rate among infants according to the NIP for protecting them against severe infections, and future vaccines should address emerging serotypes and reduce the IPD. An early diagnosis followed by an appropriate antibiotic therapy are essential in order to reduce severe complications and improve the outcomes, especially for vulnerable children.

## 6. Limitations

The study was conducted at a single center and included a small number of pediatric patients. This may reduce the statistical power and limit the ability to detect subtle epidemiological trends. Additionally, the retrospective nature of the study resulted in some data being incomplete or absent, and patients could not be monitored after discharge. Potential referral bias exists, as children from rural areas may have less access to specialized pediatric care compared to those from urban area. The data on pneumococcal serotyping distribution were limited in the earlier years of the investigation, as samples were only sent for serotyping during the final two years of the study period. Prospective, multi-center studies with a larger patient population and an extended follow-up are necessary in order to validate these observations.

## 7. Public Health Impact

Our study highlights a concerning decline in pneumococcal vaccination rates in Romania. Strengthening immunization, examining vaccine hesitancy, and ensuring better access to vaccination, particularly in rural areas, could reduce the burden of pneumococcal disease.

With the recent approval of PCV20 in the EU, policymakers should consider its potential benefits for inclusion in the NIP, since it offers a broader coverage.

## Figures and Tables

**Table 1:** Presentations versus admissions in our hospital by year.

	Emergency Dep.	Acute Therapy Dep.
2019	225,799	746
2020	20,992	363
2021	30,075	414
2022	46,459	461
2023	42,141	438
2024	47,152	398

**Table 2:** Patient’s demographics and bacterial pathogen identification results.

		No (%)
Male		38 (57.5)
Female		28 (42.5)
Age (mean, range)		5 y. o. (3 m.–17 y. o)
Romanian		27 (41.0)
Rome		23 (34.8)
Hungarian		16 (24.2)
Rural residence		38 (57.5)
Urban residence		28 (42.5)
Fully vaccinated		27 (41.0)
Partially vaccinated		20 (30.3)
Not vaccinated		19 (28.8)
Pleural fluid culture	*S. pneumoniae*	6 (9.0)
	*H. influenzae*	1 (1.5)
Pleural fluid PCR	*S. pneumoniae*	1 (1.5)
Blood culture	*S. pneumoniae*	4 (6.0)
	Coagulase (−) *Staphylococci*	5 (7.5)
	*P. aeruginosa*	1 (1.5)
	*S. pyogenes*	1 (1.5)
	*K. oxytoca*	1 (1.5)
	*K. pneumoniae*	1 (1.5)
Blood PCR	*S. pneumoniae*	3 (4.5)
	*H. influenzae*	1 (1.5)
	*S. viridans*	1 (1.5)
	*P. aeruginosa*	1 (1.5)
Laryngo-tracheal secretions	*S. pneumoniae*	4 (6.0)
	*H. influenzae*	1 (1.5)
	*P. aeruginosa*	2 (3.0)
Respiratory PCR	*S. pneumoniae*	6 (9.0)
	*M. catarrhalis*	2 (3.0)
Nasal swab	*S. pneumoniae*	3 (4.5)
	*H. influenzae*	4 (6.0)
	*S. pyogenes*	1 (1.5)
	MRSA	1 (1.5)
	*P. aeruginosa*	2 (3.0)
Oropharyngeal swab	*S. pyogenes*	1 (1.5)
	*P. aeruginosa*	1 (1.5)

## Data Availability

The original contributions presented in this study are included in the article. Further inquiries can be directed to the corresponding author.

## Abbreviations

The following abbreviations are used in this manuscript:

PCV	Pneumococcal conjugate vaccine
IPD	Invasive pneumococcal disease
PCR	Polymerase chain reaction
PPE	Parapneumonic pleural effusion
EU	European Union
RSV	Respiratory syncytial virus
ED	Emergency department
WBC	White blood cells
ESR	Erythrocyte sedimentation rate
CRP	C-reactive protein
CT	Computer tomography
MRSA	Methicillin-resistant Staphylococcus aureus
TB	Tuberculosis
NIP	National Immunization Program
